# The Baculovirus-Expressed Binding Region of *Plasmodium falciparum* EBA-140 Ligand and Its Glycophorin C Binding Specificity

**DOI:** 10.1371/journal.pone.0115437

**Published:** 2015-01-14

**Authors:** Joanna Rydzak, Radoslaw Kaczmarek, Marcin Czerwinski, Jolanta Lukasiewicz, Jolanta Tyborowska, Boguslaw Szewczyk, Ewa Jaskiewicz

**Affiliations:** 1 Ludwik Hirszfeld Institute of Immunology and Experimental therapy, Polish Academy of Sciences, Wroclaw, Poland; 2 Faculty of Physiotherapy and Physical Education, Opole University of Technology, Opole, Poland; 3 Department of Recombinant Vaccines, Intercollegiate Faculty of Biotechnology, University of Gdansk and Medical University of Gdansk, Gdansk, Poland; 4 Department of Molecular Biology, University of Zielona Góra, Zielona Góra, Poland; Ehime University, JAPAN

## Abstract

The erythrocyte binding ligand 140 (EBA-140) is a member of the *Plasmodium falciparum* DBL family of erythrocyte binding proteins, which are considered as prospective candidates for malaria vaccine development. The EBA-140 ligand is a paralogue of the well-characterized *P. falciparum* EBA-175 protein. They share homology of domain structure, including Region II, which consists of two homologous F1 and F2 domains and is responsible for ligand-erythrocyte receptor interaction during invasion. In this report we describe, for the first time, the glycophorin C specificity of the recombinant, baculovirus-expressed binding region (Region II) of *P. falciparum* EBA-140 ligand. It was found that the recombinant EBA-140 Region II binds to the endogenous and recombinant glycophorin C, but does not bind to Gerbich-type glycophorin C, neither normal nor recombinant, which lacks amino acid residues 36–63 of its polypeptide chain. Our results emphasize the crucial role of this glycophorin C region in EBA-140 ligand binding. Moreover, the EBA-140 Region II did not bind either to glycophorin D, the truncated form of glycophorin C lacking the N-glycan or to desialylated GPC. These results draw attention to the role of glycophorin C glycans in EBA-140 binding. The full identification of the EBA-140 binding site on glycophorin C molecule, consisting most likely of its glycans and peptide backbone, may help to design therapeutics or vaccines that target the erythrocyte binding merozoite ligands.

## Introduction

In the invasion of human red blood cells multiple proteins, expressed by *Plasmodium* merozoites, are involved [1, 2, 3, 4]. Two protein families play central roles in this process: the Duffy binding-like (DBL) and reticulocyte binding-like (RBL) protein family [5]. *P. falciparum* has several members of the DBL family, including erythrocyte binding-ligands (EBL), which enable the merozoite to interact with independent erythrocyte receptors and define alternative invasion pathways. Four functional *P. falciparum* EBL proteins have been identified: erythrocyte binding antigen 175 (EBA-175), erythrocyte binding antigen 140 (EBA-140), erythrocyte binding antigen 181 (EBA-181) and erythrocyte binding ligand 1 (EBL-1) [6]. All these antigens contain several conserved regions, such as Region II, which is involved in receptor binding on erythrocytes. The well-studied *P. falciparum* EBA-175 merozoite ligand [7] recognizes sialic acids present on clusters of O-linked glycans attached to glycophorin A (GPA), which is the most abundant human erythrocyte glycophorin [7, 8, 9, 10].

The merozoite ligand EBA-140 [11, 12, 13] which is released to parasite culture supernatant was shown to bind glycophorin C (GPC) [14, 15], a minor erythrocyte sialoglycoprotein [16], thus mediating a distinct invasion pathway into human erythrocytes. The EBA-140 ligand binds to erythrocytes in a sialic acid-dependent manner, similarly as EBA-175 ligand. It was proposed that the receptor for the EBA-140 ligand might be a cluster of N- and O-linked sialylated glycans on the GPC molecule [17, 18, 19]. Recently, the crystal structure of the recombinant EBA-140 erythrocyte binding region (Region II), obtained in bacteria, in a complex with a glycan containing sialic acid has been characterized and the role of individual glycan contact amino acid residues in specific sialic acid interactions was revealed [19, 20]. Since the EBA-140 ligand failed to bind the natural deletion variant of GPC Gerbich-type [15, 17, 18, 21, 22], which lacks amino acid residues 36–63 [23], it suggested that this region might be the receptor site. These results provided evidence supporting the idea that selection of Gerbich-negativity in Melanesians (Papua New Guinea) confers protection against *P. falciparum* invasion. However, the results indicating the region between the a.a. residues 13 and 22 of GPC as the binding site for EBA-140 ligand have also been shown [14]. These discrepancies highlight that the precise GPC receptor site awaits elucidation.

The major limitation in studies on the specificity of *P. falciparum* EBA ligands is their expression and purification as soluble and properly folded recombinant proteins in sufficient amounts. The recombinant whole Region II (F1 and F2 domains) or F2 domain of the homologous EBA-175 ligand were obtained using bacterial [24, 25], insect [26, 27] or yeast cells [28]. The recombinant F2 domain of the homologous EBA-175 antigen expressed in *E. coli* was purified from inclusion bodies and renatured by oxidative refolding. Taking into account that the EBA-175 Region II contains several disulphide-bridges, an yeast or baculovirus eukaryotic expression system seemed to be most suitable. The soluble, recombinant EBA-140 Region II or its F2 domain was obtained in bacteria [19, 29], *P. pastoris* [30] or in the baculovirus expression system [31]. However, the studies with the use of baculovirus-expressed EBA-140 Region II were limited to investigation of its binding to heparan sulfate proteoglycans on RBCs. Region II of the EBA-140 antigen was also expressed on the surface of Chinese hamster ovary (CHO-K1) cells [17], COS7 cells [21, 32] and HEK-293T cells [20], as a functional but insoluble, membrane-bound recombinant protein.

In this report we describe the first studies on the GPC-related specificity of the EBA-140 ligand with the use of its recombinant, baculovirus-expressed Region II. The results show that the EBA-140 ligand binds to the normal and recombinant GPC, but does not bind to the normal and recombinant Gerbich-type GPC, which lacks amino acid residues 36–63 of its polypeptide chain. Our results suggest that this GPC region and GPC oligosaccharide chains play a crucial role in the EBA-140 ligand binding.

## Materials and Methods

### Cells

The *Spodoptera frugiperda* Sf9 insect cells (ATCC) were maintained in TNM-FH (Hyclone) medium supplemented with 10% fetal calf serum (Gibco) and 25 u/ml of penicillin, 25 ug/ml of streptomycin, 62,5 ng/ml amphotericin (Antibiotic-Antimycotic Solution, Sigma-Aldrich) or in HyQ CCM3 synthetic medium (Hyclone) for expression of EBA-140 recombinant Region II. The stable clones of CHO (Hamster ovary) cells expressing GPC and its deletion mutants (Gerbich and Yus) [33] were grown in a OptiMem (Gibco) medium supplemented with 10% fetal calf serum and 100 U/ml of penicylin and 100 ug/ml of streptomycin (Pen Strep Solution, Sigma-Aldrich). Normal and Gerbich-negative human erythrocytes were freshly donated and collected on EDTA. Participants have provided their written informed consent to participate in this study. The study was approved by the Ethics Committee of Wroclaw Medical University in Wroclaw, Poland (No. 726/2011).

### Cloning of EBA-140 antigen Region II

The gene encoding Region II (amino acids 141–756) of the EBA-140 ligand (GenBank: AF 332918_1) was cloned from genomic DNA of *P. falciparum* clone Dd2 (ATCC, MR4, Manassas, VA, USA) with forward primer: CAA TAT ACG TTT ATA CAG AAA CGT ACT CAT TTG TTT GCT and reverse primer: TAT ATC GTG TTT TGT TTT AGG ATA TTT A. Polymerase Taq (Fermentas) was used in 35 cycles of amplification (94°C, 30 s; 54°C, 30 s; 72°C, 2 min 30 s) after a hot start at 94°C for 5 minutes and 10 minutes of final extension at 72°C. The PCR product was purified using a gel extraction kit (Qiagen) and cloned into a pDrive cloning vector (Qiagen) using a T4 DNA ligase (Fermentas). The resulting pDrive-RII recombinant plasmid was transformed into *E. coli* XL1 Blue competent cells and selected on LB-agar with 100 µg/ml ampicillin (Roth). The sequence was confirmed by restriction fragment analysis and DNA sequencing and used for obtaining recombinant baculovirus.

### Recombinant baculovirus expression

The recombinant baculovirus containing the EBA-140 Region II cDNA sequence coding for the protein of 632 a.a. residues, including 6xHis and c-myc tags at its C-terminus, was obtained by GeneScript (Hong Kong, China). The high titer (2x10^8^ pfu/ml) virus was inoculated into SF9 cells (3x10^6^ ml^-1^) cultured in 1 liter of CCM3 serum-free medium at a multiplicity of infection of 5. The infected cells were incubated at 27°C and 110 rpm for 2 days and a culture supernatant was collected. The recombinant Region II secreted into the medium was purified by Ni-NTA affinity chromatography.

### Purification of the recombinant EBA-140 Region II

The insect cells culture supernatant was dialysed against column buffer (50 mM Tris-HCl, 300 mM NaCl, pH 7.5) containing 5 mM β-mercaptoethanol. The precipitated material was centrifuged at 23000xg for 30 min. The supernatant was applied on 3 ml of nickel Ni-NTA resin (Qiagen) equilibrated with column buffer. The resin was loaded onto a 10 ml column washed with 100 ml of column buffer. To remove nonspecifically binding proteins, 20 ml of the column buffer containing 20 mM imidazole was used. The bound proteins were eluted with column buffer containing imidazole at the concentrations: 50 mM, 100 mM and 200 mM. Elution fractions were analyzed by SDS-PAGE and Western blotting. The fractions containing recombinant Region II were pooled and concentrated ten times using a 30,000 M_r_ cut-off Amicon Ultra device (Millipore) and then dialyzed against 50, 20 and finally 10% glycerol. The protein concentration was determined with Nanodrop (Thermo) The recombinant EBA-140 Region II was stored frozen at-80°C.

The analytical size-exclusion chromatography used for evaluation of the purified recombinant Region II (250 µg) was performed on a 10/300 GL Superdex 200 column equilibrated with column buffer containing 10% glycerin at a flow rate of 0.4 ml/min at room temperature (RT). Protein molecular weight standards used were: 66.4 kDa albumin, 43 kDa ovalbumin, 25 kDa chymotrypsinogen A, 14.4 kDa cytochrome c (Sigma-Aldrich). Protein content was evaluated spectrophotometrically at 280 nm.

### SDS-PAGE

The proteins were separated in the presence of SDS using 10% polyacrylamide gel, according to the Laemmli method [34] and visualized with Coomassie Brilliant Blue R-250 (CBB, Merck). The PageRuler Prestained Protein Ladder (Fermentas) was used as a protein standard.

### Western blotting

The proteins fractionated by SDS-PAGE were transferred to the nitrocellulose membrane (Schleicher & Schuell) according to the method of Towbin et al [35] and detected with mouse monoclonal antibody (MoAb) directed against a c-myc epitope (clone 9E10, ATCC).

### Circular dichroism of purified recombinant Region II

The folding state of the baculovirus-expressed Region II of EBA-140 antigen was evaluated by circular dichroism (CD) spectroscopy. CD spectra were recorded on a Jasco J-600 spectropolarimeter (Jasco Inc.) at room temperature with the path length of 1 mm. Each spectrum represents the average of four scans. The data are presented as mean residue molar ellipticity [θr].

### Functional analysis of the baculovirus-expressed EBA-140 Region II

**Flow cytometry analysis.** The recombinant Region II (4 ug) was incubated with 3x10^5^ native or treated with trypsin (Sigma-Aldrich) or neuraminidase (*Vibrio cholerae*, Serva) human normal or Gerbich-negative erythrocytes in phosphate buffered saline (PBS), pH 7.4 for 2 h at 4°C. Alternatively, recombinant Region II (4 ug) was incubated with 1x10^6^ CHO cells [33] expressing recombinant GPC or its deletion forms: Gerbich and Yus. After three washings with PBS, the cells were incubated for 1 hour at 4°C with rabbit serum (diluted 1:200) raised against whole Region II [29]. Then, after 3 washings with PBS, the cells were incubated for 30 min at 4°C with FITC-conjugated swine anti-rabbit Ig antibody (DakoCytomation) and analyzed for fluorescence intensity using flow cytometry (FACSCalibur, BD Biosciences).

**Immunoblotting (overlay assay).** The proteins of erythrocyte membranes or purified erythrocyte glycophorins [36] were fractionated by SDS-PAGE and transferred to nitrocellulose membrane (NC) (Schleicher & Schuel). Removal of sialic acid residues was performed by the incubation of the blots in 0.025 M surfuric acid at 80°C for 1 hour. The blots were overlayed with the solution of recombinant Region II (50ug/ml) in TBS overnight at room temperature. The bound Region II was detected on NC with a mouse monoclonal antibody (MoAb) directed against the c-myc epitope (clone 9E10, ATCC). Alternatively, GPC was detected on the blots with the MoAbs: clone 1G4 or clone 1F6 recognizing amino acids 1–4 or 110–115 of GPC, respectively [37, 38].

**Surface plasmon resonance (SPR) analysis.** The SPR assay using BIACORE T200 instrument (Biacore) was developed to analyze Region II interaction with human erythrocytes. The Ni-NTA chip (Biacore) was coated with Ni^+2^ by injection of 10 ul (0.5 nM) NiCl_2_ at a flow rate of 10ul/min. The His-tagged recombinant Region II was captured on the Ni-coated chip by injecting Region II (10ug/ml) for one minute at a flow rate of 10ul/ml. Human erythrocytes, suspended in PBS pH 7.4 containing 0.5 mg/ml BSA, of different hematocrits (0.05, 0.025, 0.0125%) were injected at flow rate of 3ul/min for 4 minutes in flow cells coated with RII. Dissociation was monitored for 300 seconds. The surface was regenerated by injecting 350 mm EDTA and 0.005% P20 surfactant at a flow rate of 30ul/min. A flow cell without treatment with Region II or NiCl_2_ was used as the control.

## Results

### Baculovirus expression of EBA-140 Region II

**Figure 1:**
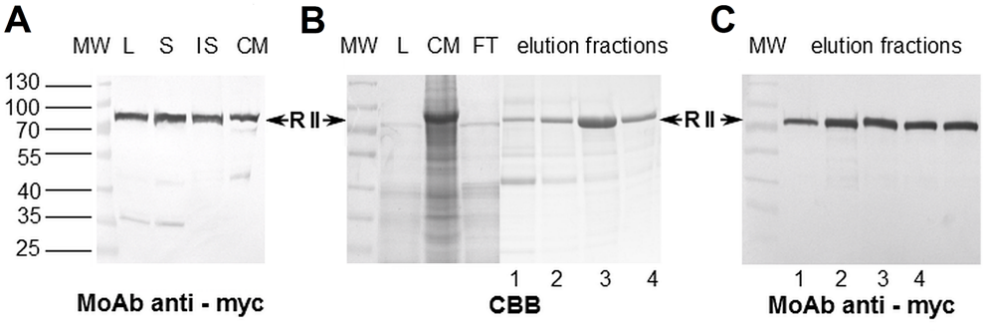
Expression and purification of the recombinant EBA-140 Region II. A)Western blotting of baculovirus-infected Sf9 cells at 50 h post-infection with MoAb anti-myc (clone 9E10); MW, protein molecular weight standards; L, cell lysate; S, soluble cell fraction; IS, insoluble cell fraction; CM, culture medium. B) Affinity purification of the recombinant EBA-140 Region II on Ni-NTA resin. SDS–PAGE of proteins in the gel stained by CBB; MW, protein molecular weight standards; L, cell lysate; CM, culture medium; FT, flow- through of resin. C) Western blotting of fractions eluted with 50mM (1,2) and 100mM (3, 4) imidazole, with MoAb anti-myc (clone 9E10); RII, Region II (~ 75 kDa).

The recombinant Region II of the EBA-140 ligand cloned from genomic DNA of Dd2 *P. falciparum* clone and was expressed in the Sf9 line of insect cells using recombinant baculovirus obtained from Genescript (titer 2x10^8^). Pilot experiments determined that 50 hours of SF9 cell culture at a virus multiplicity of infection (MOI) of 5 gave optimal expression of Region II to cell culture medium. The recombinant protein of the expected molecular mass (~75 kDa) was present in a total cell lysate and in a soluble and an insoluble cell fraction and medium fluid as well and was not degraded (Fig. 1A).

### Purification of EBA-140 Region II

**Figure 2:**
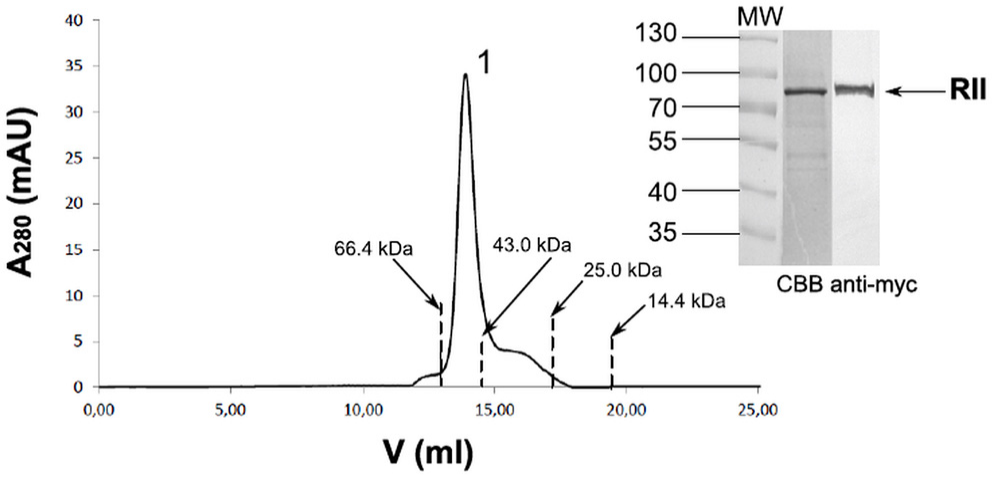
Analytical size exclusion chromatography of the EBA-140 Region II on Superdex 200 column. Purified protein was analyzed by SDS-PAGE in the gel stained with CBB and Western blotting with anti-myc MoAb (clone 9E10); peak 1 corresponds to the Region II (13,5ml elution volume); MW, protein molecular weight standards; RII, Region II (~ 75 kDa); elution volumes of standards used in column calibration are marked by the dashed lines.

The recombinant Region II expressed in 1 liter of Sf9 cell culture at the conditions described above was purified by one-step affinity chromatography on Ni-NTA resin. The fractions eluted with imidazole containing Region II were analyzed in SDS-PAGE and Western blotting (Fig. 1B,C). The fractions eluted with 100 mM imidazole were pooled and concentrated. The purity of the obtained recombinant Region II was evaluated by analytical size-exclusion chromatography (Fig. 2). The Superdex 200 elution profile indicated one peak between 66.4 and 43.0 kDa corresponding to the Region II. The molecular mass of the Region II was confirmed in SDS-PAGE and Western blotting and it was lower than expected (75 kDa) probably due to non-specific interaction between Region II molecule and carbohydrate residues of column resin. The baculovirus-expressed recombinant Region II of EBA-140 antigen was more than 95% pure and not degraded. The yield of the recombinant protein was 2–4 milligrams from one liter of Sf9 cells culture, depending on preparation.

### Conformation analysis of EBA-140 Region II

**Figure 3:**
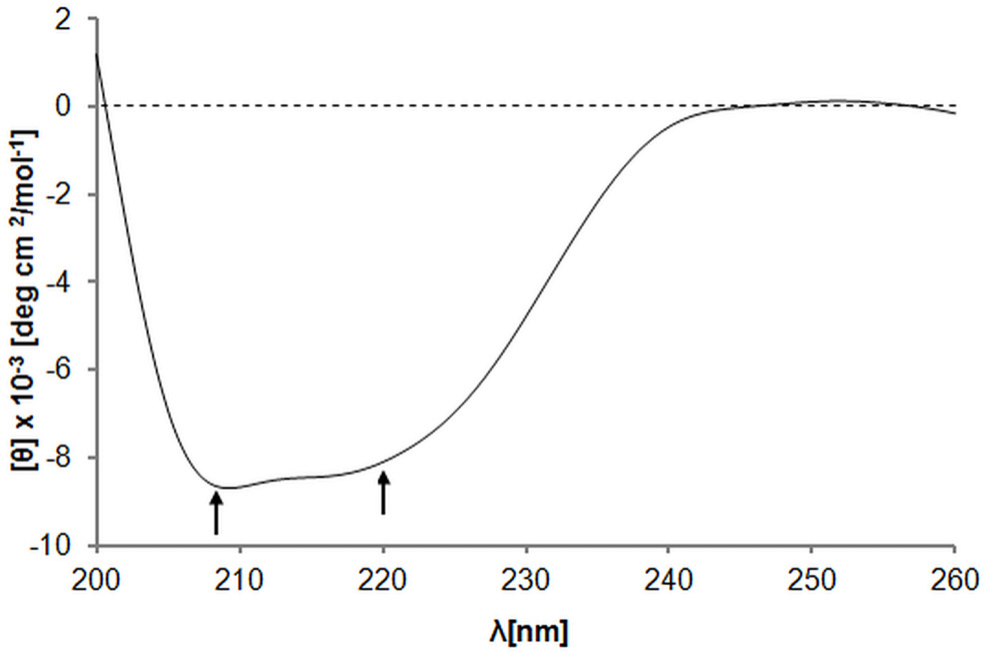
Circular dichroism spectrum (195–270 nm) of EBA-140 Region II. Solution of Region II (0.5 µM) in 50 mM Tris-HCl, 300 mM NaCl, 10% glycerin, pH 7.5 was used.

The folding state of the recombinant EBA-140 Region II was evaluated by circular dichroism (Fig. 3). The CD spectrum revealed minima near 208 and 220 nm, similar to the recombinant F2 domain of the EBA-175 homologous *P. falciparum* ligand obtained in the bacterial system [24]. These results indicate the presence of significant α-helical content in EBA-140 recombinant Region II.

### Binding of EBA-140 Region II to erythrocytes

**Figure 4:**
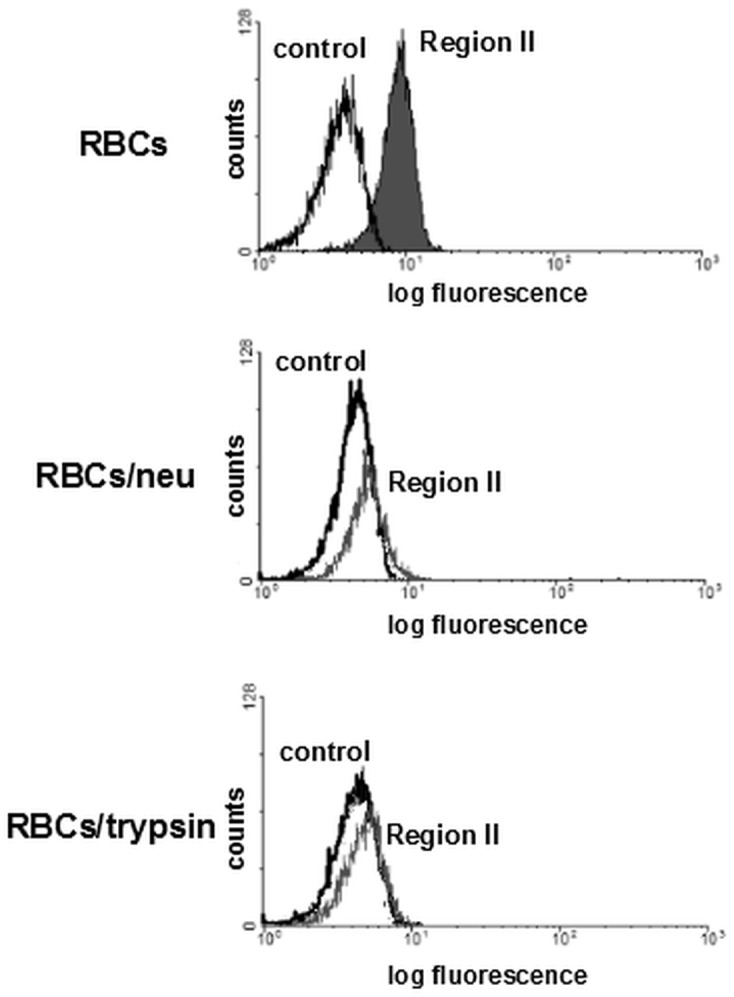
Flow cytometry analysis of EBA-140 Region II binding to the native and neuraminidase (RBCs/neu) or trypsin (RBCs/trypsin)-treated human erythrocytes (RBCs). The black line corresponds to control (RBCs incubated with the heat denatured Region II and then with anti-Region II rabbit serum); the grey line (peak) corresponds to Region II of EBA-140 antigen.

Binding of the recombinant EBA-140 Region II to human erythrocytes was evaluated by flow cytometry using native, neuraminidase or trypsin-treated red blood cells (Fig. 4). It was shown that the EBA-140 Region II binds to normal human erythrocytes, but its binding to erythrocytes treated with neuraminidase or trypsin was significantly decreased. These results indicated that baculovirus-expressed Region II is a functional protein and its interaction with human erythrocytes is specific and sialic acid-dependent. The observed decrease of RII binding to trypsin-treated erythrocytes was accordant with earlier findings that the minor sialoglycoprotein-GPC is the receptor for the EBA-140 ligand on human erythrocytes.

**Figure 5:**
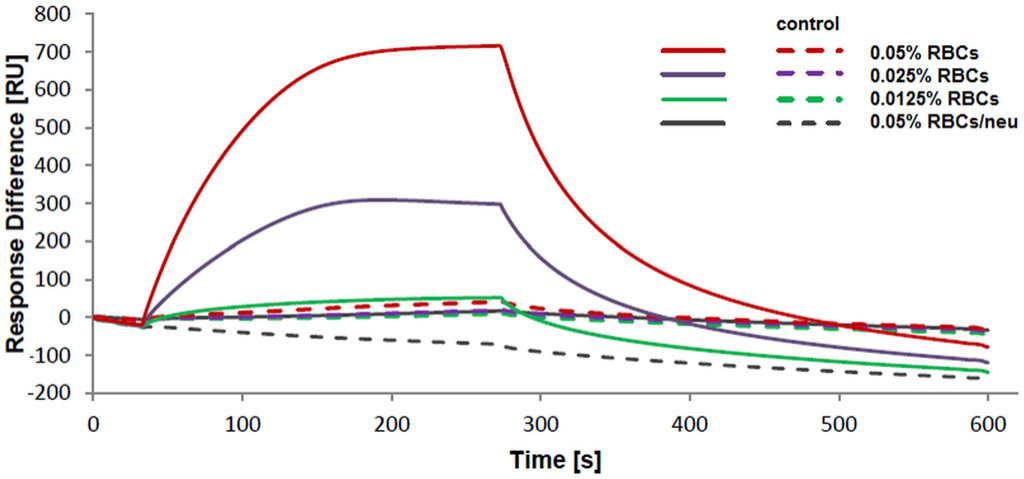
Surface plasmon resonance analysis of human RBCs binding to the recombinant EBA-140 Region II. The solid lines correspond to RBCs binding to NTA flow cell covered with the functional Region II; the dashed lines correspond to RBCs binding to NTA flow cell covered with the denatured RII; analysis was performed in PBS buffer with 0.5% BSA, pH 7.2 on Biacore T200.

To study the interaction of the recombinant Region II with human red blood cells the surface plasmon resonance (SPR) based assay using Biacore T200 was also developed (Fig. 5). The binding of erythrocytes to RII-coated wells on a NTA chip was measured. The response difference was dependent on erythrocyte hematocrit and reached ~700 RU at 0.05% hematocrit for normal erythrocytes but was significantly decreased for neuraminidase treated erythrocytes. This is consistent with the flow cytometry results shown in Fig. 4. In summary, the flow cytometry and SPR assays indicate that the binding of the recombinant Region II with human erythrocytes is dependent on sialic acid.

### Binding of EBA-140 Region II to glycophorin C

**Figure 6:**
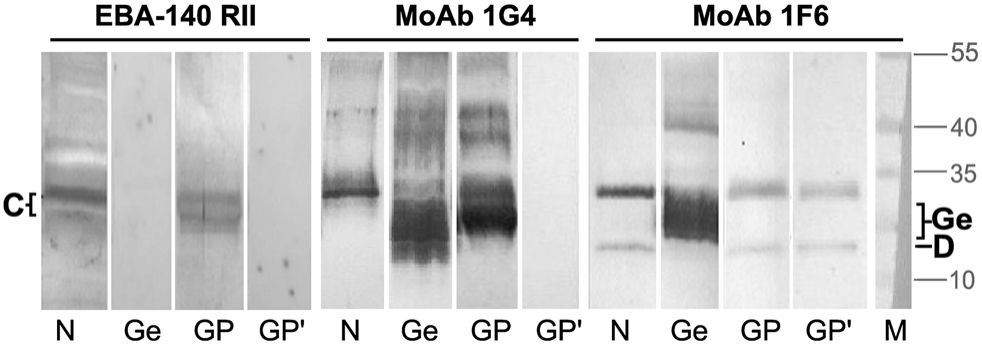
The binding of EBA-140 Region II to GPC in Western blotting. Normal (N), Gerbich (Ge) RBCs membranes and purified RBCs glycophorins (GP) were fractionated in SDS-PAGE and transferred to NC. RBCs glycophorins were desialylated (GP’) by the incubation of the blots in sulfuric acid. The blots were overlaid with the solution of recombinant Region II detected with MoAb anti-myc (clone 9E10). The GPC, GPD and variant GPC Gerbich was detected with MoAbs specific to N-terminus (clone 1G4) or C-terminus (clone1F6) of GPC [37, 38]. GPC is visible on the blots as a double band stained with MoAb 1G4 or a single band stained with MoAb 1F6 due to a partial degradation of GPC at the C-terminus; M, protein molecular weight standards.

The binding specificity of the recombinant *P. falciparum* EBA-140 ligand was preliminarily characterized in immunoblotting using human erythrocyte membranes and purified glycophorins [36] (Fig. 6). Recombinant Region II of EBA-140 ligand binding to normal erythrocyte membranes (N) revealed two bands, one corresponding to GPC and the lower band which is supposed to be partially degraded GPC or might be a different protein. Clearly, Region II fails to bind glycophorin D (GPD), the truncated form of GPC lacking a.a. residues 1–21 [39], and deletion variant GPC type Gerbich lacking a.a. residues 36–63 [40]. Moreover, the EBA-140 Region II binding to GPC was abolished after desialylation of the receptor on the blot.

**Figure 7:**
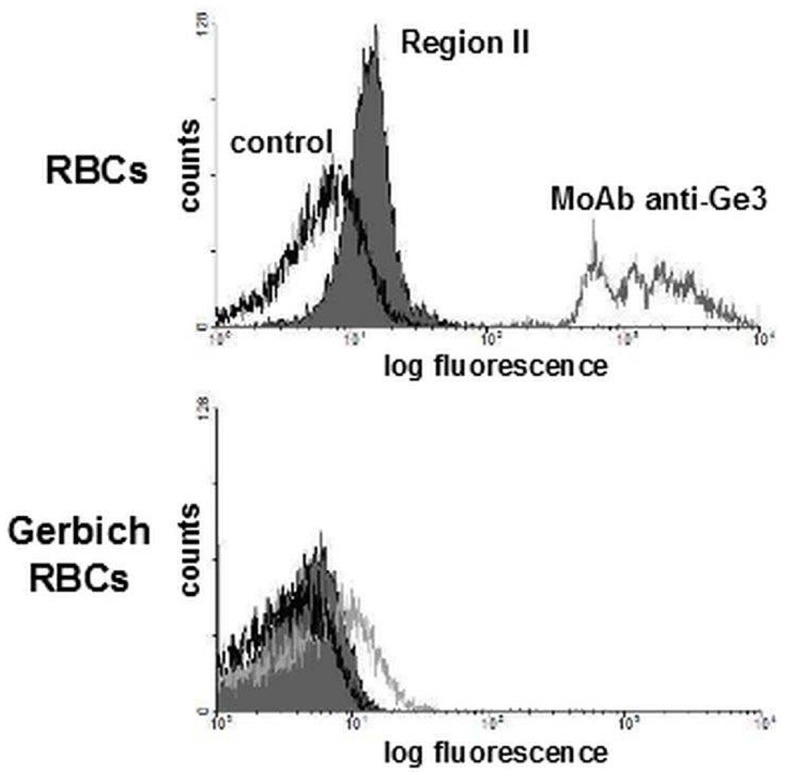
Flow cytometry analysis of EBA-140 Region II binding to normal and variant human RBCs Gerbich phenotype containing a deletion variant of GPC (Δ36–63 a.a.). The black line corresponds to the control (RBCs incubated with the heat denatured Region II and then with anti-Region II rabbit serum); the grey peak corresponds to the binding of Region II to RBCs; the grey line corresponds to RBCs binding of MoAb anti-Ge3 recognizing a.a. residues 43–51 of GPC (clone 3C4) [38], indicating normal or Gerbich-negative RBCs phenotype.

The binding of recombinant Region II to freshly donated normal and Gerbich-negative erythrocytes was examined using flow cytometry (Fig. 7). The inability of recombinant Region II to bind to Gerbich-negative erythrocytes indicates that GPC region comprising amino acids 36–63 is crucial for EBA-140 ligand receptor recognition.

**Figure 8:**
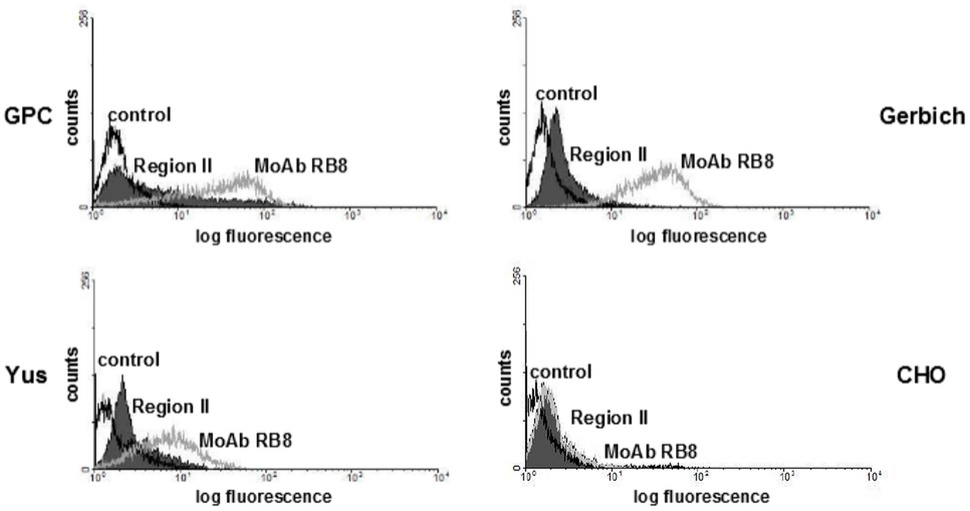
Flow cytometry analysis of EBA-140 Region II binding to normal and variant recombinant forms of GPC (Yus and Gerbich) expressed in CHO cells. The black line corresponds to the control (CHO cells incubated with anti-Region II rabbit serum); the grey peaks correspond to the binding of Region II to transfected or untransfected CHO cells; the grey line corresponds to the binding of MoAb RB8, recognizing a.a. residues 13–17 of GPC [37], to recombinant forms of GPC [33].

Recombinant Region II binding was also determined using CHO cells stable transfected with vectors encoding GPC and its deletion forms type Yus and Gerbich [33] (Fig. 8). The CHO cells showed the relatively weak but similar expression of the recombinant forms of GPC. However, the EBA-140 Region II showed weak but distinct binding to GPC expressing cells and no binding to those expressing Gerbich-type GPC. The data concerning GPC Yus-type are less conclusive, because the expression of GPC Yus on CHO cells was still lower than that of normal and Gerbich GPC. Indeed, comparing the binding of the EBA-140 Region II to Yus- and Gerbich-type GPC expressing cells indicates a binding of this ligand to GPC Yus lacking amino acids 17–36.

Recombinant Region II binding was also determined using CHO cells stable transfected with vectors encoding GPC and its deletion forms type Yus and Gerbich [33] (Fig. 8). The CHO cells showed the relatively weak expression of the recombinant forms of GPC. However, the EBA-140 Region II showed weak but distinct binding to GPC expressing cells, clear binding to GPC Yus expressing cells and no binding to those expressing Gerbich-type GPC. In summary, the binding of the EBA-140 Region II to Yus-type GPC expressing CHO cells indicates that this ligand with GPC Yus lacking amino acids 17–36.

## Discussion

The erythrocyte-binding antigen 140 (EBA-140) is a *Plasmodium falciparum* erythrocyte invasion ligand that engages GPC on host erythrocytes [14, 15, 17, 18, 22]. To demonstrate direct interaction between EBA-140 and GPC culture supernatants from parasites were used [14, 15, 22]. Alternatively, EBA-140 erythrocyte binding domain- (Region II) was transiently expressed on the surface of COS7 cells [21] or CHO-K1 cells [17]. Studies based on the expression of the recombinant EBA-140 Region II on the cell surface suggested that polymorphism within this ligand found for different strains of *P. falciparum* alters the receptor binding specificity. The GPC specificity of EBA-140 ligand was defined by only one sequence (VSTK) of four Region II polymorphic amino acid residues [17, 21]. However, another research group showed that these polymorphisms had a quantitative effect affected the level of the EBA-140 binding to GPC, but not its receptor specificity [22].

Our results were generally accordant with earlier findings obtained with parasite culture supernatants or with the recombinant EBA-140 Region II expressed on cells, but were for the first time obtained with the purified baculovirus-expressed recombinant Region II of the EBA-140 ligand. The ligand was shown to bind to erythrocyte and recombinant form of GPC and no distinct binding to other erythrocyte membrane components was observed. The role of GPC oligosaccharide chains in this reaction was confirmed by the lack of the EBA-140 Region II binding to desialylated GPC and much lower binding to desialylated erythrocytes. It was shown that the EBA-140 Region II was not bound either to Gerbich deletion mutant of GPC (lacking a.a. residues 36–63) or to GPD (truncated GPC lacking a.a. residues 1–21), but showed the binding to the recombinant Yus variant of GPC (lacking a.a. residues 17–35).

**Figure 9:**
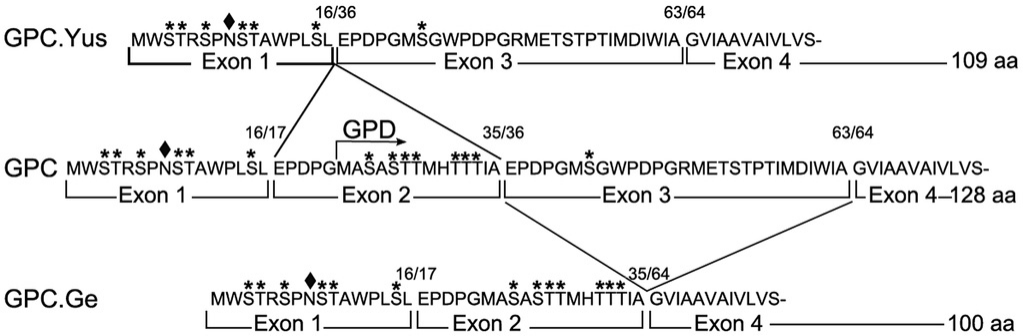
Structure of N-terminal fragments of the polypeptide chain of GPC and its deletion variants Yus and Gerbich type. Asterisks denote O- glycosylation sites, the black oval denote the N-glycan. The numbers of a.a. residues relate to their position in normal GPC. The location of regions coded by exons: 1, 2, 3, 4 is indicated.

In view of these results the binding site on GPC molecule for the EBA-140 ligand is still a puzzle. GPC has many sialylated oligosaccharide chains, one N-glycan bound to Asn8 and several O-linked glycans [16] (Fig. 9) and their role in the EBA-140 ligand binding is generally accepted. It was reported that the GPC N-glycan is essential for the EBA-140 binding, but the role of individual O-glycans cannot be ruled out [18]. The loss of binding activity by desialylation not always indicates a direct engagement of sialic acid residues in the binding. Desialylation may have a conformational effect which makes peptidic binding site unavailable [41, 42]. However, a recently obtained crystal structure of the EBA-140 Region II in complex with a sialolactose revealed two glycan pockets, one in each DBL domain [20]. It supports the conclusion that GPC sialoglycan participate directly in the interaction with EBA-140 antigen. The lack of EBA-140 ligand binding to similarly glycosylated glycophorin A (GPA) strongly suggests that GPC protein backbone also contributes to the binding, either direcly (glycopeptidic binding site), or by providing a proper exposure of glycans. Therefore, it seems logical that the EBA-140 binding site is located within the richly O-glycosylated and N-glycan containing N-terminal portion of GPC, that is in agreement with the lack of the EBA-140 Region II binding to GPD. However, the problem is complicated by the lack of the EBA-140 Region II binding to Gerbich deletion variant of GPC ([15, 18, 21, 22] and this report). The question arises which is the role of a.a. residues 36–63 of GPC, lacking in GPC Gerbich. An essential role of this region is supported by the observation that the Gerbich-negative phenotype is present at a high frequency in Papua New Guinea, where infection with *P. falciparum* is common [15]. This fragment of GPC is distant from the N-terminal portion and contains only one putative O-glycan (Fig. 9) therefore it is unlikely that this domain may provide O-glycans that could interact directly with the EBA-140 ligand. More likely, the lack of this domain might affect the secondary structure of GPC molecule, which influences the accessibility of N-glycan and (or) the other O-glycans attached to GPC domains coded by exons 1 and 2. Unfortunately, this hypothesis is not supported by our results showing the lack of the EBA-140 Region II binding to Gerbich-negative erythrocyte membranes in Western blotting assay, where denatured and thus fully accessible regions of variant GPC polypeptide chain were examined.

There is another possibility that the lack of the EBA-140 binding to Gerbich variant of GPC is the result of different structure of its N-glycosidic chain. Unlike normal GPC the variant GPC Gerbich-type migrate as a diffuse band on SDS-PAGE (Fig. 6). Studies using endoglycosydases suggested that the diffuse nature of GPC-Gerbich band results from carbohydrate heterogeneity and that the variant GPC contain N-glycosidic chain with repeating lactosamine units [43]. It was proposed that this altered N-glycosylation of GPC-Gerbich might be a result of the better aviability of the truncated molecule for membrane-bound glycosyltransferases. The different sugar composition of GPC-Gerbich N-glycan was also shown by Miller group [18]. The N-linked oligosaccharide of variant GPC contained an elevated proportion of mannose suggesting the presence of high Man-type structures in addition to sialylated complex-type oligosaccharide chains. These authors argued that the difference in the N-glycan structure and the failure to inhibit the binding of the EBA-140 ligand to erythrocytes with de-N-glycosylated GPC suggested that its N-glycosidic chain is critical for binding of the EBA-140 ligand.

We recently performed the first mass spectrometry analysis of the GPC N-glycan structure which revealed the presence of a small portion of complex-type structures containing repeating polylactosamine units with the terminal Fucα(1,2) residues, while the short antenna were terminated with Siaα(2,6) residues. Based on these results we propose that the amount of elongated, Fuc-terminated polylactosamine chains may be elevated in GPC-Gerbich, in agreement with previous results [43]. This difference might explain the lack of the EBA-ligand binding to Gerbich-negative erythrocytes due to variant GPC N-glycan fucosylation and indicates the crucial role of the sialic acid residues attached to GPC N-glycosidic chain in ligand-receptor interaction. The full understanding of molecular interactions between the EBA-140 ligand and its GPC receptor [44] on human erythrocytes might be important in respect to the design of *P. falciparum* invasion inhibitory therapeutics and vaccines.
